# Combined albumin-bilirubin grade and Mac-2 binding protein glycosylation isomer as a useful predictor in compensated liver cirrhosis

**DOI:** 10.1097/MD.0000000000018366

**Published:** 2019-12-16

**Authors:** Hiroki Nishikawa, Hirayuki Enomoto, Kazunori Yoh, Yoshinori Iwata, Yoshiyuki Sakai, Kyohei Kishino, Naoto Ikeda, Tomoyuki Takashima, Nobuhiro Aizawa, Ryo Takata, Kunihiro Hasegawa, Noriko Ishii, Yukihisa Yuri, Takashi Nishimura, Hiroko Iijima, Shuhei Nishiguchi

**Affiliations:** a Division of Hepatobiliary and Pancreatic disease, Department of Internal Medicine; b Center for Clinical Research and Education, Hyogo College of Medicine, Nishinomiya, Hyogo, Japan.

**Keywords:** albumin-bilirubin, FIB4 index, liver cirrhosis, Mac-2 binding protein glycosylation isomer, prognostic model

## Abstract

We aimed to compare the impact on survival among albumin-bilirubin (ALBI) grade, modified ALBI (mALBI) and our proposed combined ALBI grade and Mac-2 binding protein glycosylation isomer (M2BPGi) or FIB4 index grading system in chronic hepatitis C (CHC) related compensated liver cirrhosis (n = 165, 93 men and 72 women, median age = 67 years). Patients with ALBI grade 1, 2, and 3 were allocated a score of 1, 2, and 3 points, respectively. Patients with mALBI grade 1, 2A, and 2B were allocated a score of 1, 2, and 3 points, respectively. Patients with a high or low M2BPGi were allocated a score of 1 and 0 point. Patients with a high or low FIB4 index were allocated a score of 1 and 0 point. Sum of the point of ALBI (1, 2, or 3) and M2BPGi (0 or 1) or FIB4 index (0 or 1) was defined as ALBI-M2BPGi grade or ALBI-FIB4 grade. Prognostic accuracy was compared using the Akaike information criterion (AIC) value and time dependent receiver operating characteristics (ROC) curve analysis. The median follow-up duration was 5.422 years. AIC value for survival by ALBI-M2BPGi grade was the lowest among 4 prognostic models (AIC: 205.731 in ALBI grade, 200.913 in mALBI grade, 189.816 in ALBI-M2BPGi grade, and 204.671 in ALBI-FIB4 grade). All area under the ROC curves of ALBI-M2BPGi grade in each time point were higher than those of ALBI grade, mALBI grade, and ALBI-FIB4 grade. In conclusion, our proposed ALBI-M2BPGi grading system seems to be helpful for estimating prognosis in patients with CHC related compensated LC.

## 1 Introduction

Liver cirrhosis (LC) is an end-stage status in chronic liver injury caused by several factors such as hepatitis virus, alcohol abuse, and autoimmune disorders, etc.^[1–3]^ Prognosis in compensated LC progressively worsens with the cumulative occurrence of ascites, variceal hemorrhage, hepatic encephalopathy (HE), hepatocellular carcinoma (HCC), and spontaneous bacterial peritonitis.^[1–3]^ Prognostic model in patients with compensated LC is therefore clinically of importance for the better prediction of survival.

The major limitation of the Child-Pugh scoring system is that it includes several subjective parameters (HE and ascites) and interrelated parameters (ascites and serum albumin).^[4]^ Ascites can be easily influenced by diuretic use or dehydration state. Diagnosing minimal or covert HE involves difficulties. To overcome these limitations, a simple assessment method for liver functional reserve, called albumin-bilirubin (ALBI) grade, which is based on 2 objective parameters (serum albumin level and total bilirubin level), has been recently proposed.^[5]^ The predictability of ALBI grade has been confirmed for patients with LC with or without HCC irrespective of liver disease etiologies.^[6–12]^ Predicting clinical outcomes in patients with LC is challenging and is likely best accomplished with a combination of objective parameters in addition to the clinical course of LC. Thus, combined ALBI grade and other objective data grading systems have been proposed for the better predictive accuracy over ALBI grade.^[13–18]^

Recently, a novel liver fibrosis marker (Mac-2 binding protein glycosylation isomer [M2BPGi]), which is a glycobiomarker associated with chronic hepatitis C (CHC)-related liver fibrosis with a unique fibrosis-related glycoalteration, has been established by Japanese investigators.^[19–21]^ The usefulness of M2BPGi for the prediction of the severity of liver fibrosis has been well validated, while FIB4 index is also a well validated liver fibrosis marker in patients with CHC.^[22–26]^ While for hepatitis B virus infection HCC can occur at any time even in the absence of fibrosis, for hepatitis C virus (HCV), there is a strong association between LC and HCC.^[1,3]^ The FIB4 index is therefore more of a proxy for evaluating advanced liver fibrosis (which in turn is associated with a higher risk of HCC), rather than a true, direct indicator of HCC.^[24]^

However, there have been no reports examining the impact of combined ALBI grade and M2BPGi or FIB4 index on clinical outcomes in patients with LC. In this study, we sought to compare the predictive accuracy on survival among ALBI grade, modified ALBI (mALBI), and our proposed combined ALBI grade and M2BPGi or FIB4 index grading system in patients with compensated LC.

## 2 Patients and methods

### 2.1 Patients

A total of 165 individuals with CHC-related compensated LC (available stored serum samples in all patients) were admitted to our hospital between March 2007 and June 2015, and they were subjected to this analysis. Compensated LC indicated Child-Pugh A LC. Subjects with CHC-related liver disease were defined as those with HCV antibody positive and hepatitis B surface antigen negative. LC was determined based on pathologic findings, radiologic findings, and/or laboratory data.^[27–30]^ M2BPGi was tested as reported elsewhere using stored serum samples.^[31]^ FIB4 index was calculated as reported previously.^[32]^

During the follow-up period after the LC diagnosis, blood biochemical and radiological tests with the aim of identifying cancer incidence or LC-related complications were periodically performed (at 3–6 months interval). When serum albumin level showed <3.5 g/dL, nutritional supplementation therapies were considered.^[33]^ Antiviral treatments such as direct acting antivirals (DAAs) or interferon-based treatment regimens were also considered.^[33]^ In principal, diagnosis for HCC and strategies for HCC therapy were determined according to the current guidelines.^[34,35]^

### 2.2 ALBI score, ALBI grade, and mALBI grade

ALBI score in each subject was calculated as reported previously.^[5]^ Patients with ALBI grade 1, 2, and 3 were allocated a score of 1, 2, and 3 points, respectively. Patients with mALBI grade 1, 2A, and 2B were allocated a score of 1, 2, and 3 points, respectively.^[36]^

### 2.3 M2BPGi, FIB4 index, ALBI-M2BPGi grade, and ALBI-FIB4 grade

The median M2BPGi value in our study was 5.29 cutoff index (COI). High and low M2BPGi was defined as M2BPGi value ≥5.29 COI and <5.29 COI. Patients with a high M2BPGi were allocated a score of 1, whereas patients with a low M2BPGi were allocated a score of 0. The median FIB4 index in our study was 4.90. High and low FIB4 index was defined as FIB4 index ≥4.90 and <4.90. Patients with a high FIB4 index were allocated a score of 1, whereas patients with a low FIB4 index were allocated a score of 0. Sum of the point of ALBI (1, 2, or 3) and M2BPGi (0 or 1) or FIB4 index (0 or 1) was defined as ALBI-M2BPGi grade or ALBI-FIB4 grade. ALBI-M2BPGi grade and ALBI-FIB4 grade therefore ranged from 1 to 4. We compared the predictive ability for survival among ALBI grade, mALBI grade, ALBI-M2BPGi grade, and ALBI-FIB4 grade.

This study protocol was acknowledged by the institutional review board in Hyogo college of medicine (approval no. 1831) and all clinical investigations were done in compliance with the Declaration of Helsinki. All patients gave written informed consent.

### 2.4 Statistical analyses

Quantitative variables were compared by Pearson correlation coefficient or Spearman rank correlation, as appropriate. Survival curves in ALBI grade, mALBI grade, ALBI-M2BPGi grade, and ALBI-FIB4 grade were made by the Kaplan–Meier method and compared in the log-rank test. Akaike information criterion (AIC) with each assessment method was tested for comparison of survival. The fitness of the models was compared based on AIC and the lowest value of AIC provided the best fit to the data. Furthermore, we analyzed time-dependent receiver operating characteristics (ROC) curves of ALBI grade, mALBI grade, ALBI-M2BPGi grade, and ALBI-FIB4 grade for survival and compared area under the ROCs (AUCs) among these 4 assessment methods in each time point (2-, 3-, 4-, 5-, 6-, and 7- year).^[32,37,38]^ Data were shown as median value (range). The significance threshold in this analysis was *P*< .05 using the statistical analysis software (JMP 14 [SAS Institute Inc., Cary, NC]).

## 3 Results

### 3.1 Baseline characteristics

**Figure 1:**
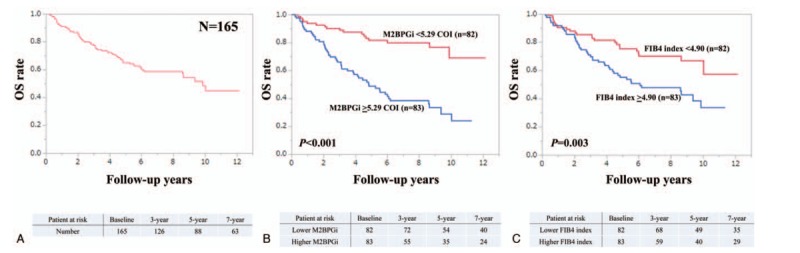
(A) Cumulative overall survival rate for all cases (n = 165). (B) Kaplan–Meier curves in patients with M2BPGi ≥5.29 cutoff index (COI) and <5.29 COI. (C) Kaplan–Meier curves in patients with FIB4 index ≥4.90 and <4.90. M2BPGi = Mac-2 binding protein glycosylation isomer.

Demographic and clinical characteristics of the analyzed subjects (n = 165) were demonstrated in Table 1. The study cohort included 93 men and 72 women with the median age (range) of 67 (23–93) years. The median follow-up duration was 5.422 years. There were 48 patients (29.1%) with ALBI grade 1, 117 patients (70.9%) with ALBI grade 2, and none with ALBI grade 3. While there were 48 patients (29.1%) with mALBI grade 1, 49 patients (29.7%) with mALBI grade 2A, 68 patients (41.2%) with mALBI grade 2B, and none with mALBI grade 3. M2BPGi ranged from 0.66 COI to 19.95 COI (median, 5.29 COI). FIB4 index ranged from 0.86 to 33.27 (median, 4.90). The 3-, 5-, and 7-year cumulative overall survival (OS) rates for all cases were 77.93%, 64.95%, and 58.56% (Fig. 1A). Patients with high M2BPGi had significantly lower OS rate than those with low M2BPGi (*P* < .001, Fig. 1B). Likewise, patients with high FIB4-index had significantly lower OS rate than those with low FIB4-index (*P* = .003, Fig. 1C). HCC was identified at baseline in 68 cases (41.2%). During the follow-up period, sustained virological response (SVR) was achieved in 83 patients (50.3%) by antiviral treatments. Of these, 34 patients were treated with interferon-based antiviral treatments and the remaining 49 patients were treated with interferon-free DAA treatments.

**Table 1:**
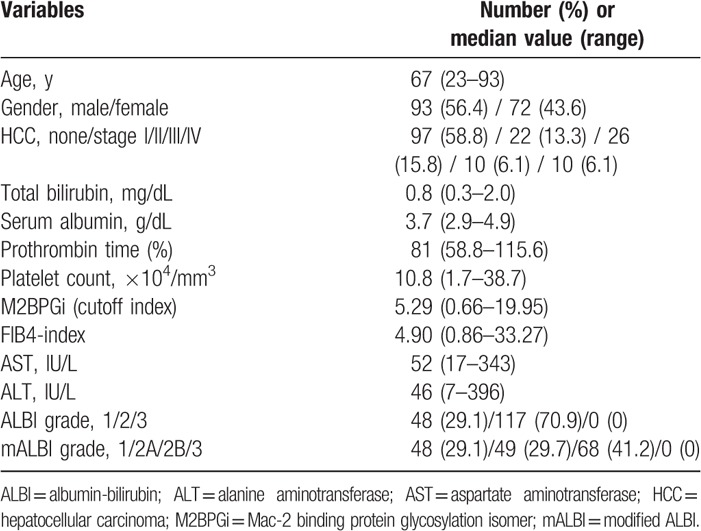
Baseline characteristics (n = 165).

### 3.2 Patient numbers according to ALBI-M2BPGi grade and ALBI-FIB4 grade

There were 38 patients with ALBI-M2BPGi grade 1, 54 patients with ALBI-M2BPGi grade 2, 73 patients with ALBI-M2BPGi grade 3, and none with ALBI-M2BPGi grade 4. There were 33 patients with ALBI-FIB4 grade 1, 64 patients with ALBI-FIB4 grade 2, 68 patients with ALBI-FIB4 grade 3, and none with ALBI-FIB4 grade 4.

### 3.3 Causes of death

During the observation period, 69 patients (41.8%) died. The causes of death were hepatic failure in 27 patients, advanced HCC status in 31, and other causes in 11.

### 3.4 Comparison of prognostic accuracy among 4 assessment methods for all cases

**Figure 2:**
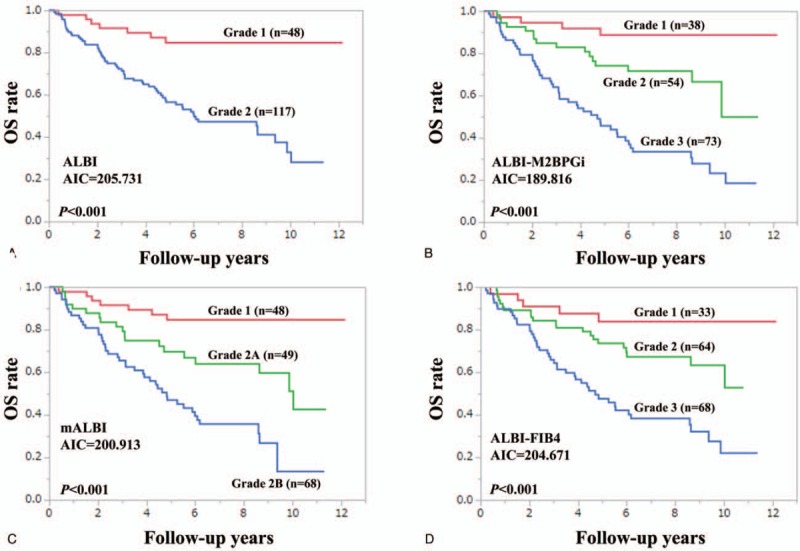
Kaplan–Meier curves according to ALBI grade (A), ALBI-M2BPGi grade (B), mALBI (C), and ALBI-FIB4 (D) for all cases. ALBI = albumin-bilirubin, M2BPGi = Mac-2 binding protein glycosylation isomer, mALBI = modified ALBI.

Patient survival was well stratified by ALBI grade (*P* < .001), mALBI grade (*P* < .001), ALBI-M2BPGi grade (*P* < .001), and ALBI-FIB4-index (*P* < .001) for all cases. We compared predictive accuracy among 4 prognostic models for all cases. The AIC value for survival by ALBI-M2BPGi grade was the lowest among 4 prognostic models (AIC: 205.731 in ALBI grade, 200.913 in mALBI grade, 189.816 in ALBI-M2BPGi grade, and 204.671 in ALBI-FIB4 grade) (Fig. 2A–D).

### 3.5 Comparison of prognostic accuracy among 4 assessment methods in patients with or without HCC

**Figure 3:**
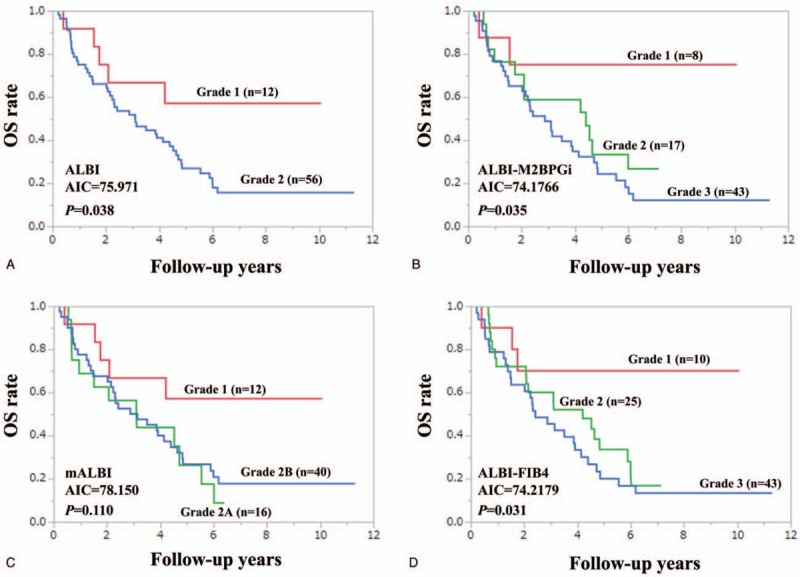
Kaplan–Meier curves according to ALBI grade (A), ALBI-M2BPGi grade (B), mALBI (C), and ALBI-FIB4 (D) in HCC cases (n = 68). ALBI = albumin-bilirubin, HCC = hepatocellular carcinoma, M2BPGi = Mac-2 binding protein glycosylation isomer, mALBI = modified ALBI.

**Figure 4:**
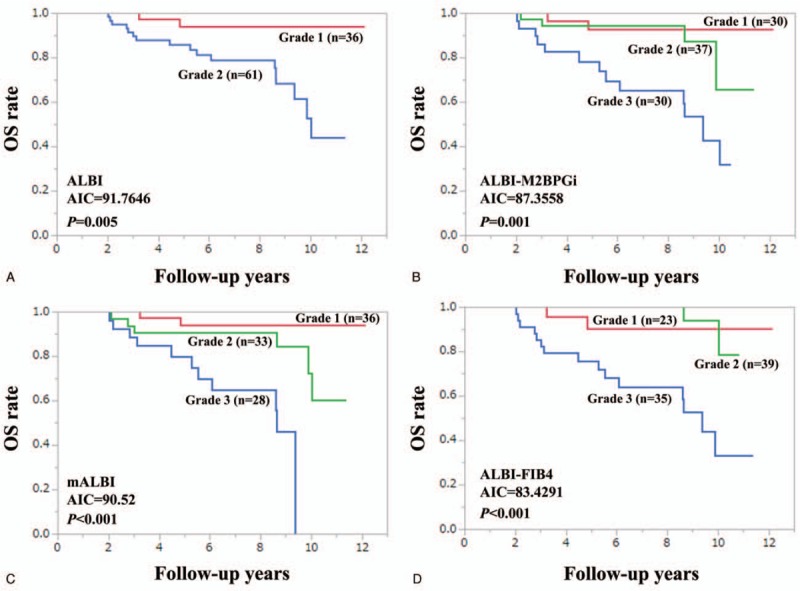
Kaplan–Meier curves according to ALBI grade (A), ALBI-M2BPGi grade (B), mALBI (C), and ALBI-FIB4 (D) in non-HCC cases (n = 97). ALBI = albumin-bilirubin, HCC = hepatocellular carcinoma, M2BPGi = Mac-2 binding protein glycosylation isomer, mALBI = modified ALBI.

In patients with HCC (n = 68), we compared predictive accuracy among 4 prognostic models. The model with the lowest AIC was ALBI-M2BPGi grade (AIC = 74.1766), followed by ALBI-FIB4 grade (AIC = 74.2179) (Fig. 3A–D). Similarly, in patients without HCC (n = 97), we compared predictive accuracy among 4 prognostic models. The model with the lowest AIC was ALBI-FIB4 grade (AIC = 83.4291), followed by ALBI-M2BPGi grade (AIC = 87.3558) (Fig. 4A–D).

### 3.6 Comparison of prognostic accuracy among 4 prognostic models using time-dependent ROC analysis

**Figure 5:**
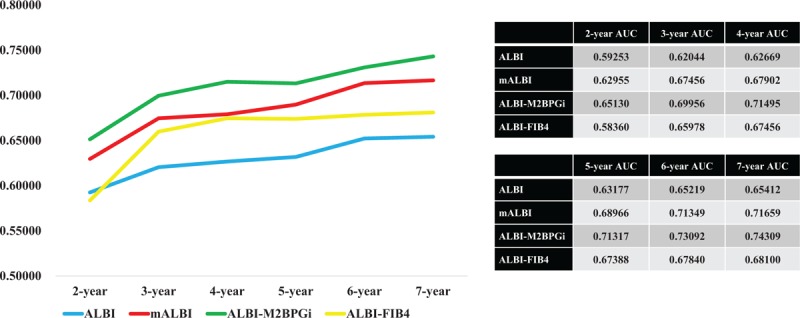
Time-dependent ROC analyses at 2-, 3-, 4-, 5-, 6- and 7-year of ALBI grade, mALBI grade, ALBI-M2BPGi grade, and ALBI-FIB4 grade for all cases. ALBI = albumin-bilirubin, M2BPGi = Mac-2 binding protein glycosylation isomer, mALBI = modified ALBI, ROC = receiver operating characteristics.

Results for time-dependent ROC analyses at 2-, 3-, 4-, 5-, 6-, and 7-year of ALBI grade, mALBI grade, ALBI-M2BPGi grade, and ALBI-FIB4 grade for all cases were shown in Fig. 5. All AUCs of ALBI-M2BPGi grade in each time point were higher than those of ALBI grade, mALBI grade, and ALBI-FIB4 grade, indicating that ALBI-M2BPGi grade had superior predictive ability for survival over other prognostic models.

### 3.7 Correlation between ALBI score and M2BPGi and FIB4 index for all cases

**Figure 6:**
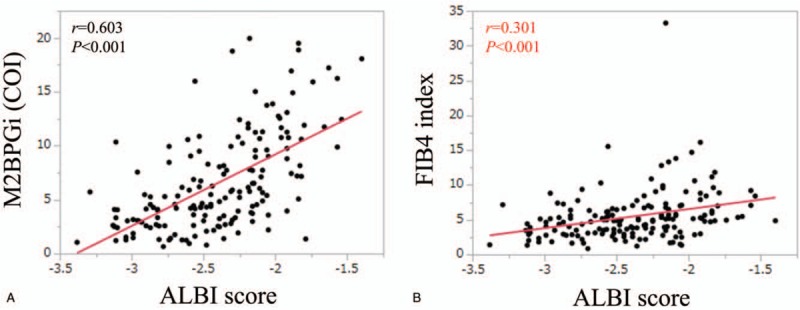
Correlation between ALBI score and M2BPGi (A) and FIB4 index (B). ALBI = albumin-bilirubin, M2BPGi = Mac-2 binding protein glycosylation isomer.

M2BPGi significantly correlated with ALBI score (*r* = 0.603, *P* < .001). (Fig. 6A)

FIB4 index also significantly correlated with ALBI score (*r* = 0.301, *P* < .001). (Fig. 6B).

## 4 Discussion

ALBI grade is a simple objective evaluation method. A lot of clinical studies have verified the superior predictive accuracy of ALBI grade over the Child-Pugh classification.^[6–12]^

M2BPGi and FIB4 index are currently acknowledged as useful liver fibrosis markers in CHC patients.^[19–26]^ ALBI grade does not incorporate liver fibrosis markers and we hypothesized that collaboration of these markers has potential to exert higher predictability for patients with compensated LC. In the current study, we demonstrated the superior predictive accuracy of our proposed ALBI-M2BPGi grading system over ALBI grade, mALBI grade, and ALBI-FIB4 grade not only by comparing AICs among 4 assessment methods but also using ROC analysis with a consideration of time dependence.

Time-dependent ROC analysis can be helpful for estimating the predictability of markers.^[38]^ Most studies for evaluating OS involve a long-time follow-up interval and the status of a subject (dead or alive) is updated at each time point in time-dependent ROC curve analysis.^[38]^ In our data, AUCs in all time points of ALBI-M2BPGi grade was consistently higher than those of ALBI grade, mALBI grade, and ALBI-FIB4 grade, indicating the favorable predictability of our proposed grading system. The significant correlation between ALBI score and M2BPGi level (*r* = 0.603, *P* < .001) may be linked to our current results. The synergistic effect of ALBI and M2BPGi on OS may be underlying.

Recently, the advent of oral DAA agents has dramatically improved SVR rates for HCV therapy, providing SVR rates >95% with a favorable safety profile and shorter treatment period.^[39,40]^ In this study, SVR during the follow-up period was identified in 83 patients (50.3%) and 14 patients have survived for >10 years at the time of analysis. Increased serum albumin level and decreased liver fibrosis markers level can be observed in HCV patients with SVR.^[41]^ Nevertheless, ALBI-M2BPGi grade had the lowest AIC among 4 assessment methods for all cases and for HCC patients, suggesting the robustness of our proposed grading system. Whereas 1 weak point is that the impact of ALBI-M2BPGi was diminished in patients without HCC. In patients without HCC, the model with the lowest AIC was ALBI-FIB4 grade, although the reasons for these are unclear.

ALBI grade 2 includes subjects with a wide range of liver functional reserve as well as those rated as Child-Pugh B and thus in the modified version of ALBI grade, ALBI grade 2 was divided into 2 subtypes (2A and 2B).^[36]^ In our data, as demonstrated in Fig. 2C, 2 subtypes were well stratified, while in patients with HCC, such results were not obtained (Fig. 3C). Presence of HCC may be linked to our results.

Several limitations should be acknowledged in this analysis. First, this is a retrospective and single center observational study and the usefulness of our proposed ALBI-M2BPGi grading system should be verified in other independent cohorts. Secondly, our study subjects were limited to patients with CHC-related compensated LC; whether our proposed grading system could be extrapolated to patients with compensated or decompensated LC with other liver disease etiologies requires additional investigation. However, our study results denoted that ALBI-M2BPGi can be a helpful grading system. In conclusion, we identified ALBI-M2BPGi grade as the strongest ability to separate patients with compensated LC into different prognostic groups. Our proposed ALBI-M2BPGi grading system seems to be helpful for estimating prognosis in patients with compensated LC.

## Acknowledgments

The authors would like to thank all medical staff in our hospital for their support.

## Author contributions

**Data curation:** Hiroki Nishikawa, Hirayuki Enomoto, Kazunori Yoh, Yoshinori Iwata, Yoshiyuki Sakai, Kyohei Kishino, Naoto Ikeda, Tomoyuki Takashima, Nobuhiro Aizawa, Ryo Takata, Kunihiro Hasegawa, Noriko Ishii, Yukihisa Yuri, Takashi Nishimura, Hiroko Iijima.

**Formal analysis:** Hiroki Nishikawa.

**Methodology:** Hiroki Nishikawa, Hirayuki Enomoto.

**Supervision:** Shuhei Nishiguchi.

**Writing – original draft:** Hiroki Nishikawa.

**Writing – review & editing:** Hirayuki Enomoto, Shuhei Nishiguchi.
